# Patient-reported outcome measures for life engagement in mental health: a systematic review

**DOI:** 10.1186/s41687-022-00468-5

**Published:** 2022-06-10

**Authors:** Roger S. McIntyre, Zahinoor Ismail, Christopher P. Watling, Catherine Weiss, Stine R. Meehan, Primrose Musingarimi, Michael E. Thase

**Affiliations:** 1 Mood Disorders Psychopharmacology Unit, University Health Network, University of Toronto, 399 Bathurst Street, MP 9-325, Toronto, ON M5T 2S8 Canada; 2 Departments of Psychiatry, Clinical Neurosciences, and Community Health Sciences, Hotchkiss Brain Institute and O’Brien Institute for Public Health, University of Calgary, Calgary, AB Canada; 3 Cambridge—a Prime Global Agency, Cambridge, UK; 4 Otsuka Pharmaceutical Development & Commercialization Inc., Princeton, NJ USA; 5 H. Lundbeck A/S, Valby, Copenhagen, Denmark; 6 Perelman School of Medicine, University of Pennsylvania and the Philadelphia Veterans Affairs Medical Center, Philadelphia, PA USA

**Keywords:** Depression, Mood disorders, Anhedonia, Cognition, Emotional blunting, Function, Motivation, Life engagement, Mental health, Patient reported outcome measures

## Abstract

**Background:**

Life engagement in the context of mental health is a broad term that describes positive health aspects relating to cognition, vitality, motivation and reward, and the ability to feel pleasure—concepts that are meaningful to patients. The aim of this systematic literature review was to identify validated patient-reported outcomes (PROs) that can assess any aspect of life engagement in adults, in the field of general mental health.

**Methods:**

This was a systematic literature review of articles in English from the MEDLINE database (date of search: September 9, 2020). The search strategy had three components: (1) terms to capture PROs; (2) terms to capture mental health; and (3) terms to capture aspects of life engagement. Articles were eligible if they included a PRO that: (1) is named; (2) can be used across mental health disorders; (3) is used to assess any aspect of life engagement; and (4) has undergone psychometric validation and/or qualitative content validation. A list of PROs was extracted.

**Results:**

A total of 1585 records were screened and 233 articles were eligible for inclusion. Within these 233 articles, 49 distinct PROs were identified, two of which specifically captured their authors’ interpretation of life engagement: the Engaged Living Scale (ELS) and the Life Engagement Test (LET). However, while the ELS and LET covered motivation and reward, life fulfillment, and value-based living, neither scale captured the cognitive or vitality aspects of life engagement. The remaining identified PROs generally captured single aspects of life engagement, most commonly motivation/reward/energy–apathy, pleasure–anhedonia, and mental/psychological well-being.

**Conclusion:**

Numerous PROs are available that may capture aspects of life engagement. However, a need remains for a new PRO that can be used in clinical trials to provide a more comprehensive description of the improvements in life engagement that patients with mental health disorders may experience with successful treatment.

## Background

A patient’s subjective view of their own health is increasingly being recognized as an essential part of health care. Patient-reported outcomes (PROs) can be used in clinical research to capture outcomes that are meaningful to the patient, in a manner that is standardized across the trial population [1, 2]. The United States Food and Drug Administration (FDA) encourages the use of patient experience data, including PROs, in clinical trials [3], and both the FDA and European Medicines Agency (EMA) are developing guidance to advance patient-focused drug development [4, 5]. PROs can also be used in clinical practice to monitor patient progress and to assess treatment benefits [2].

In mental health, there are numerous PROs that can measure patient well-being, quality of life, treatment satisfaction, patient needs, and the therapeutic relationship [6–8]. There remains a need for PROs that can measure other concepts that are meaningful to patients, such as ‘life engagement’.

In the context of mental health, a conceptual framework for patient life engagement has been developed by Weiss et al., with the objective of better defining and capturing meaningful patient functional outcomes including life fulfillment, well-being, and participation in valued and meaningful activities [9, 10]. The life engagement concept includes positive health aspects relating to cognition (including ‘hot’ cognition, i.e., cognition colored by emotion), vitality, motivation and reward, and the ability to feel pleasure, and was developed based on transcripts from patient interviews and discussion with a panel of expert psychiatrists [9]. The rationale for developing the framework was that health care professionals and patient call centers had provided unsolicited feedback to a drug manufacturer describing benefits of a depression treatment that were not fully captured by existing terminology. Specifically, in the new framework, life engagement is split into four domains: emotional (affect/mood), physical (energy), social (interest), and cognitive (alertness/thinking). The framework was validated in a separate study, in which 20 patients with major depressive disorder (MDD) were interviewed and asked to describe how they felt when engaged with life [11]. The patients’ definitions and experiences were consistent with the four domains of Weiss et al., suggesting that the framework successfully captures the concept of life engagement, and that the concept resonates with patients. In an online survey, patients with mental health disorders (depression or bipolar disorder) listed aspects of life engagement, such as being proactive, returning to activities that they used to enjoy, and being able to maintain concentration on activities, among the top indicators of treatment effectiveness [12].

The concept of life engagement overlaps with, but is not fully captured by, other concepts in mental health. For example, life engagement incorporates improvement of anhedonia (which comprises motivational and consummatory aspects [13]) and apathy (which comprises behavior/cognition, emotion, and social interaction dimensions [14])—important treatment targets in mental health [15, 16]. However, anhedonia and apathy alone do not describe the aspects of life engagement relating to attention, alertness, and clarity of thought [9]. Similarly, quality of life (defined by the World Health Organization as an individual’s perception of their position in life in the context of the culture and value systems in which they live and in relation to their goals, expectations, standards and concerns [17]) and mental well-being (which refers to an individual’s ability to develop their potential, work productively and creatively, build strong and positive relationships with others and contribute to their community [18]) do not fully capture the energy and alertness aspects of life engagement.

It is not clear if any PROs exist that are fit for purpose to measure life engagement in clinical trials or in clinical practice. The aim of this systematic literature review (SLR) was to identify validated PROs that can assess any aspect of life engagement in adults, in the field of general mental health, in order to answer the question: can the life engagement framework be evaluated in research and/or clinical practice using existing tools?

## Methods

An SLR was conducted to search for eligible PROs relating to life engagement. The search strategy and inclusion criteria were specified in advance and documented in a protocol.

### Eligibility criteria

Reports were considered eligible if they described studies in adults in any mental health area, including mental health symptoms in a non-clinical sample. The outcomes of each study must have included a PRO that (1) was named (i.e., not a one-off questionnaire or visual analogue scale); (2) could be used across mental health disorders (i.e., it was not for a specific disease); (3) was used to assess any aspect of life engagement; and (4) had undergone psychometric validation (e.g., criterion validity, construct validity) and/or qualitative content validation [19, 20]. Any study type, with or without an intervention and comparator, was permitted. Reports must have been published in English.

### Search strategy

The search strategy had three components: (1) terms to capture articles that include one or more PROs; (2) terms to capture articles in general mental health; and (3) terms to capture articles covering life engagement. The terms to capture life engagement were developed in pairs such that each aspect of life engagement had both a positively and negatively valenced descriptor (e.g., engaged–disengaged; pleasure–anhedonia; motivated–aimless). Studies were identified by searching the MEDLINE/PubMed database (which covers the period, 1964–present) using the following terms: (“patient-report*” OR “patient-rated” OR “self-report*” OR “self-rated” OR “self-evaluation”) AND (scale OR measure OR instrument OR tool) AND (“mental health” OR psychiatry) AND (engag*[title] OR motivat*[title] OR reward*[title] OR enthusias*[title] OR passion*[title] OR effort[title] OR pleasur*[title] OR “well-being”[title] OR energiz*[title] OR arous*[title] OR connected*[title] OR attent*[title] OR alert*[title] OR calmness[title] OR calm[title] OR ataraxi*[title] OR disengag*[title] OR aimless[title] OR apath*[title] OR laziness[title] OR lazy[title] OR anhedoni*[title] OR disaffection[title] OR letharg*[title] OR disconnected*[title] OR indifferen*[title] OR inattent*[title] OR excit*[title] OR ruminat*[title] OR unsettl*[title] OR initiative[title] OR ambitio*[title] OR relax*[title] OR incent*[title] OR passive[title] OR disinterest*[title] OR detach*[title]). During development of the protocol, the search strategy was tested to confirm that it captured two known PROs (the Temporal Experience of Pleasure Scale and the Snaith–Hamilton Pleasure Scale).

No language or date restrictions were applied to the electronic search, and all article types were eligible. The search was performed on September 9, 2020, by CPW.

### Study selection

First, retrieved records were screened based on their titles and abstracts, and excluded if: (1) not in English; (2) in a non-mental health topic; (3) in children or adolescents; (4) it was clear from the abstract that the article did not contain a PRO; (5) it was clear from the abstract that the article contained only a disease-specific PRO or PRO for a non-life engagement topic.

Second, full-text articles were retrieved and excluded if: (1) the article did not contain a PRO, or contained only unnamed PROs; (2) the article contained only disease-specific PROs and/or PROs for non-life engagement topics, or where questions were about a specific task/activity (e.g., sport). The scope of the SLR was full scales, not subscales or individual items. With regard to ‘well-being’, this was applied in the context of positive mental health (i.e., psychological and social well-being) [21], as opposed to physical well-being/health. Satisfaction with life was not included in the search terms, but these PROs were included because satisfaction with life is related to the concept of life engagement.

Eligibility assessment was performed by a single reviewer (CPW).

### Data extraction

PRO names were extracted from eligible articles by a single reviewer (CPW). Based on this shortlist of PROs, further research was conducted and articles were excluded if: (1) no psychometric or qualitative content evaluation was available for the PRO; or (2) no English language version of the PRO was available.

Since the purpose of the review was to generate a list of PROs, no study outcomes were extracted and no bias assessment was necessary. The number of eligible articles in which each PRO appeared was counted.

## Results

**Fig. 1:**
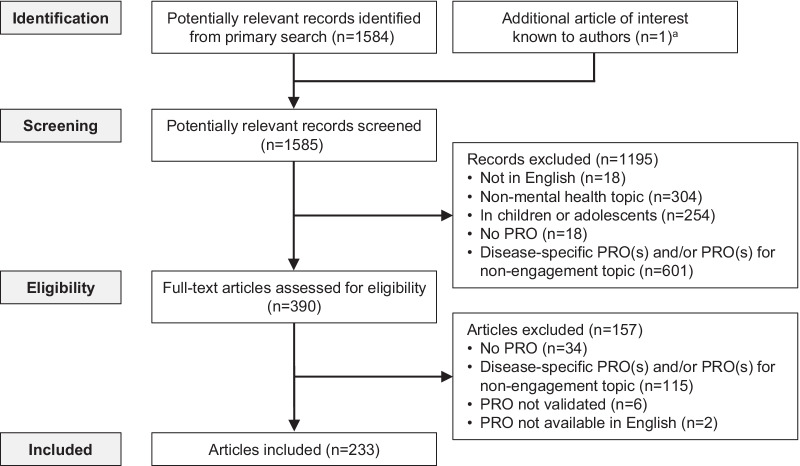
PRISMA flow diagram. ^a^This article, describing the Life Engagement Test, was not picked up by the search terms due to a lack of detail in the article’s title and abstract. PRO: patient-reported outcome

A total of 1585 records were screened, and 233 articles were eligible for inclusion (Fig. 1). Within these 233 articles, a total of 49 distinct PROs were identified (Table 1). In addition, the SLR identified two prior reviews of ‘well-being’ scales (using a very broad definition of well-being that included quality of life, physical well-being, and health), which found 99 and 60 PROs respectively [6, 7].

**Table 1:** Validated PROs to assess any aspect of patient life engagement in mental health (adults)

Concept/theme	Name of PRO [primary source]	Number of hits^a^
Life engagement	Engaged Living Scale (ELS) [22]	1
	Life Engagement Test (LET) [23]	1
Work/occupation engagement/vigor	Utrecht Work Engagement Scale (UWES) [24]	7
	Short version (UWES-9) [25]	
	Profiles of Occupational Engagement in Severe mental illness—Productive occupations (POES-P) [26]	3
	Shirom–Melamed Vigor Measure (SMVM) [27]	1
Motivation/reward/energy–apathy	Behavioral Activation System (BAS) scale [28]	28
	Apathy Evaluation Scale Self-report (AES-S) [29]	14
	Short version [30, 31]	
	Motivation and Pleasure Scale—Self-Report (MAP-SR) [32]	7
	Sensitivity to Reward (SR) scale [33]	4
	Rewarding Events Inventory (REI) [34]	3
	Motivation and Energy Inventory (MEI) [35]	1
	Environmental Reward Observation Scale (EROS) [36]	1
	Motivational Trait Questionnaire (MTQ) [37]	1
	Reward Responsiveness (RR) [38]	1
	Work Extrinsic and Intrinsic Motivation Scale (WEIMS) [39]	1
	MacCarthy Task Motivation Scale (MCTMS) [40]	1
Pleasure–anhedonia	Revised Chapman Physical and Social Anhedonia Scales (PAS/SAS) [41–43]	36
	Snaith–Hamilton Pleasure Scale (SHAPS) [44]	33
	Temporal Experience of Pleasure Scale (TEPS) [45]	29
	Fawcett–Clarke Pleasure Capacity Scale (FCPCS) [46]	6
	Anticipatory and Consummatory Interpersonal Pleasure Scale (ACIPS) [47]	5
	Hedonic Deficit & Interference Scale (HDIS) [48]	2
	Dimensional Anhedonia Rating Scale (DARS) [49]	2
	Domains of Pleasure Scale (DOPS) [50]	1
	Specific Loss of Interest and Pleasure Scale (SLIPS) [51]	1
Attention/alertness	Attentional Control Scale (ACS) [52]	14
	Mindful Attention Awareness Scale (MAAS) [53]	3
	Everyday Life Attention Scale (ELAS) [54]	2
	Emotional Attentional Control Scale (eACS) (adaptation of ACS) [55]	1
	Toronto Hospital Alertness Test (THAT) [56]	1
Connectedness/social well-being	Keyes Social Well-Being (SWB) scale [57]	3
	Social Connectedness Scale—Revised (SCS-R) [58]	2
	Autonomy–Connectedness Scale, 30-item version (ACS-30) [59]	1
Mental/psychological well-being	Warwick–Edinburgh Mental Well-Being Scale (WEMWBS) [60]	16
	Short version (SWEMWBS) [61]	
	WHO (Five) Well-Being Questionnaire (WHO-5) [62]	14
	Ryff’s scales of Psychological Well-Being (PWB) [63]	12
	Mental Health Continuum Short Form (MHC-SF) [64, 65]	3
	Flourishing Scale (FS) [66]	3
	Comprehensive Inventory of Thriving (CIT) [67]	1
	Psychological General Well-Being Index (PGWBI) [68]	1
	Inventory of General Life Functioning (GLF) (adaptation of PGWBI) [69]	1
Life satisfaction/meaning	Satisfaction With Life Scale (SWLS) [70]	16
	Personal Well-being Index (PWI) [71, 72]	3
	Meaning in Life Questionnaire (MLQ) [73]	2
Calmness–arousal	Stress Arousal Checklist (SACL) [74]	1
Rumination	Ruminative Response Scale (RRS) [75]	20
	Revised version [76]	
	Ruminative Thought Style Questionnaire (RTSQ) [77]	4
	Leuven Adaptation of the Rumination on Sadness Scale (LARSS) [78]	1
	Mini Cambridge–Exeter Repetitive Thought Scale (Mini-CERTS) [79]	1

*PRO* patient-reported outcome

^a^I.e., the number of eligible articles in which the PRO appeared

### General themes

Of the 49 identified PROs, two specifically captured their respective authors’ interpretation of life engagement (Engaged Living Scale and Life Engagement Test). In addition, three PROs captured engagement in the context of work/occupations. The remaining PROs generally captured single aspects of life engagement, rather than multiple aspects; most commonly motivation/reward/energy–apathy, pleasure–anhedonia, and mental/psychological well-being (Table 1).

The three most frequently identified PROs were specific measures of pleasure/anhedonia: the Revised Chapman Physical and Social Anhedonia Scales (appeared in 36 eligible articles), the Snaith–Hamilton Pleasure Scale (33 articles), and the Temporal Experience of Pleasure Scale (29 articles).

Many PROs appeared in eligible articles just once (20/49; 40.8%). The majority of PROs appeared ≤ 3 times (32/49; 65.3%).

### Descriptions of selected PROs

Of the 49 identified PROs, the ten judged by the authors as most relevant to life engagement are described in more detail below. These ten scales (an arbitrary number) were singled out for more detailed descriptions because they include items that can measure multiple aspects of life engagement.

#### E﻿ngaged Living Scale (ELS)﻿

The ELS [22] measures engaged living in the context of acceptance and commitment therapy (ACT). ACT is a form of cognitive behavioral therapy that aims to enhance a person’s acceptance of negative emotions, memories, and thoughts, so that they might engage in valued life activities. The ELS comprises 16 items across two subscales: Valued living (ten items, e.g., “I have values that give my life more meaning”, “I know what motivates me in life”); and Life fulfillment (six items, e.g., “I live the way I always intended to live”, “I am satisfied with how I live my life”). Items are scored on a 5-point scale, from 1 (completely disagree) to 5 (completely agree). A total score and subscale scores are obtained by summing the item scores.

In a general non-clinical sample, and in a sample with chronic pain, the ELS showed good internal consistency (Cronbach’s α = 0.90–0.91) and construct validity, correlating with outcomes including psychological well-being (Pearson’s *r* = 0.35–0.64), anxiety (*r* = − 0.30), and depression (*r* = − 0.46) [22]. The ELS has not been used in a sample with mental health disorders.

#### Life Engagement Test (LET)

The LET [23] measures purpose in life. It comprises six items assessing the extent to which a person considers their activities to be valuable and important, e.g., “There is not enough purpose in my life”, “To me, the things I do are all worthwhile”. Items are scored on a 5-point scale, from 1 (strongly disagree) to 5 (strongly agree); some items are reverse scored. A total score is obtained by summing the item scores.

In a general non-clinical sample, university students, and a variety of clinical (non-mental health) samples, the LET showed acceptable internal consistency (Cronbach’s α = 0.72–0.87), moderately stable test–retest reliability (*r* = 0.61–0.76), and construct validity based on correlations with other psychosocial measures including depression (*r* = − 0.33 to − 0.49) and mental health (*r* = 0.32–0.49) [23].

#### Shirom–Melamed Vigor Measure (SMVM)

The SMVM [27] measures vigor or energetic feelings at work during the past 30 days. It comprises 14 items across three subscales: Physical strength (five items, e.g., “I feel full of pep”, “I feel energetic”); Cognitive liveliness (five items, e.g., “I feel mentally alert”, “I feel I can think rapidly”); and Emotional energy (four items, e.g., “I feel able to show warmth to others”, “I feel able to be sensitive to the needs of coworkers and customers”). Items are scored on a 7-point scale, from 1 (never or almost never) to 7 (always or almost always).

In a sample of employees, the SMVM subscales each showed good internal consistency (Cronbach’s α = 0.95 for Physical strength, 0.72 for Cognitive liveliness, and 0.88 for Emotional energy), with moderate intercorrelation between subscales (mean *r* = 0.44) [27]. Each subscale negatively correlated with a measure of emotional exhaustion (burnout) (mean *r* = − 0.17), and positively correlated with personal accomplishment (mean *r* = 0.47) and another measure of work engagement (*r* = 0.45–0.66) [27, 80]. The SMVM has not been used in a sample with mental health disorders.

#### Apathy Evaluation Scale Self-report (AES-S)

The AES-S [29] measures apathy including interests, productivity and initiative during the past 4 weeks. The original AES-S had 18 items; subsequent factor analysis identified an Apathy subscale comprising 12 items, e.g., “Are you interested in things?”, “Is it important for you to get things done during the day?”, “Do you feel motivated?” [29–31]. Items are scored on a 5-point scale, from 0 (not at all) to 4 (very much) [29–31].

In a sample that included healthy participants and those with MDD, the AES-S showed satisfactory internal consistency (Cronbach’s α = 0.86) and test–retest reliability (Pearson’s *r* = 0.76); convergent validity with clinician-rated apathy (*r* = 0.72) (the correlation was smaller with informant-rated apathy, *r* = 0.43); divergent validity with patient-rated depression and anxiety (*r* = 0.42 for both); and was able to discriminate between healthy participants and those with MDD [29]. The AES has also been validated in psychosis/schizophrenia, and is able to distinguish patients with schizophrenia from healthy controls [31, 81].

#### Motivation and Pleasure Scale—Self-Report (MAP-SR)

The MAP-SR [32] measures motivation and pleasure during the past week. It has 15 items, covering the domains of social and recreational or work pleasure (six items, e.g., “In the past week, how often have you experienced pleasure from being with other people?”, “Looking ahead to being with other people in the next few weeks, how much pleasure do you expect you will experience from being with others?”); feelings and motivations about close, caring relationships (three items, e.g., “When it comes to relationships with your family members, how important have these relationships been to you over the past week?”); and motivation and effort to engage in activities (six items, e.g., “In the past week how motivated have you been to be around other people and do things with them?”, “In the past week how much effort have you made to actually do things with other people?”). Items are scored on a 5-point scale, from 0 (none/not at all) to 4 (very/extremely).

In schizophrenia/schizoaffective disorder, the MAP-SR showed excellent internal consistency (Cronbach’s α = 0.90), good convergent validity with another rating of motivation and pleasure (*r* = 0.65), and divergent validity from depressive/anxiety symptoms (*r* = 0.06) and positive symptoms (*r* = 0.11), though not from agitation/mania (*r* = 0.41) [32].

#### Motivation and Energy Inventory (MEI)

The MEI [35] measures vitality in the past 4 weeks. It was designed to be responsive to treatment effects and able to discriminate among patient populations with differing clinical characteristics. It comprises 27 items across three subscales: Mental energy (ten items, e.g., “During the past 4 weeks, how often did you feel satisfied with what you accomplished during the day?”, “During the past 4 weeks, how often did you have trouble getting out of bed in the morning because you didn’t want to face the day?”), Social motivation (ten items, e.g., “During the past 4 weeks, how often did you avoid social conversations with others?”, “During the past 4 weeks, how much of the time did you prefer to be alone?”), and Physical energy (seven items, e.g., “During the past 4 weeks, how often did you feel enthusiastic when you began your day?”, “During the past 4 weeks, how often did you run out of energy before the end of the day?”). Items are variably scored on a 5-, 6- or 7-point scale; some items are reverse scored.

In depression, the three MEI subscales each showed good internal consistency (Cronbach’s α = 0.75–0.89), construct validity (moderate correlation with quality of life, *r* = − 0.42 to − 0.64, and modest correlation with depressive symptoms, *r* = − 0.15 to − 0.26), and could detect changes over time [35]. In schizophrenia, an 18-item inpatient version of the MEI distinguished between patients and controls, and correlated with symptoms including depressive symptoms (Spearman’s *r* = − 0.49) and overall psychopathology (*r* = − 0.37), but not positive symptoms (*r* = − 0.14), indicating good divergent validity [82]. The 18-item inpatient version has also been used in MDD [83].

#### Dimensional Anhedonia Rating Scale (DARS)

The DARS [49] measures desire/interest, motivation, effort, and pleasure “right now”. It comprises 17 items across four domains: Hobbies/past-times, Food/drinks, Social activities, and Sensory experiences. For each domain, the patient must provide two examples of what they find rewarding (e.g., their two favorite hobbies/past-times), and then answer standardized questions about these examples (e.g., “I would enjoy these activities”, “I would spend time doing these activities”). Items are scored on a 5-point scale, from 0 (not at all) to 4 (very much); some items are reverse scored. A total score is obtained by summing the item scores.

In patients with MDD/bipolar depressive episodes, the DARS showed excellent internal consistency (Cronbach’s α = 0.96), convergent validity with another anhedonia scale (*r* = − 0.79), moderate correlation with a depression measure (*r =* − 0.37), and was able to distinguish MDD from healthy controls [49].

#### World Health Organization (Five) Well-Being Questionnaire (WHO-5)

The WHO-5 [62] measures psychological well-being over the past 2 weeks. It comprises five items: “I feel cheerful and in good spirits”, “I feel calm and relaxed”, “I feel active and vigorous”, “I wake up feeling fresh and rested”, and “My daily life is filled with things that interest me”. Items are scored on a 6-point scale, from 0 (at no time) to 5 (all the time). A ‘raw score’ from 0 to 25 is obtained by summing the item scores, and a ‘standardized percentage score’ from 0 to 100 is calculated by multiplying the raw score by 4.

The WHO-5 is a widely administered PRO that can be used irrespective of underlying illness (or lack of illness), and has been thoroughly validated across many different mental health settings [84–87]. It can be used as a screening tool for depression (a raw score below 13 is an indication that the individual should be tested for depression), and it can capture improvement in well-being over time in clinical trials [62, 85].

#### Comprehensive Inventory of Thriving (CIT)

The CIT [67] is a comprehensive measure of psychological well-being designed for use in research purposes and for in-depth assessment of well-being in health settings, such as psychiatric and clinical practices. It is also available as a brief version for initial assessment of well-being. The CIT comprises 54 items across seven dimensions: Relationships (e.g., “There are people I can depend on to help me”), Engagement (e.g., “I get fully absorbed in activities I do”), Mastery (e.g., “I use my skills a lot in my everyday life”), Autonomy (e.g., “Other people decide most of my life decisions”), Meaning (e.g., “My life has a clear sense of purpose”), Optimism (e.g., “I am optimistic about my future”), and Subjective well-being (e.g., “I feel negative most of the time”). Items are scored on a 5-point scale, from 1 (strongly disagree) to 5 (strongly agree); some items are reverse scored.

In a general non-clinical sample, the CIT subscales showed good internal consistency (Cronbach’s α = 0.71–0.96), good test–retest reliability (*r* = 0.57–0.83), convergent validity with other measures of psychological well-being, divergent validity with measures of ill-being, and good predictive validity for health and quality of life [67]. The CIT also negatively correlates with measures of depression (*r* = − 0.46) and anxiety (*r* = − 0.34) [88].

#### Stress Arousal Checklist (SACL)

The SACL [74] measures stress and arousal. It comprises two subscales, which do not correlate with each other [74, 89]. Originally, the Stress subscale consisted of 19 adjectives (tense, worried, apprehensive, bothered, uneasy, dejected, uptight, nervous, distressed, fearful, jittery, peaceful, relaxed, cheerful, contented, pleasant, comfortable, calm, restful), and the Arousal subscale consisted of 15 adjectives (active, energetic, vigorous, alert, lively, activated, stimulated, aroused, drowsy, tired, idle, sluggish, sleepy, somnolent, passive). However, other studies have since adjusted the list of adjectives (for example, 20- and 30-adjective versions are available) [90, 91]. Each adjective is rated as one of four options: ‘definitely feel’ (++), ‘feel slightly’ (+), ‘do not understand or cannot decide’ (?), and ‘definitely do not feel’ (−). These scores are dichotomized to a 2-point scale, from 0 (do not feel [− and ?]) to 1 (do feel [++ and +]) (some items are reverse scored), and summed for each subscale.

The SACL subscales are able to differentiate psychiatric inpatients from healthy controls [90]. In university students, compared with another measure of stress, the Stress subscale showed convergent validity (*r* = 0.85) and the Arousal subscale showed divergent validity (*r* = − 0.25) [89]. Compared with a measure of relaxation, the Arousal subscale showed greater correlation (*r* = 0.35) than the Stress subscale (*r* = 0.23) [91].

## Discussion

In this review, 49 validated PROs of varying complexity were identified that may be used to assess aspects of life engagement in adults with mental health symptoms. From this list, only two PROs specifically captured their respective authors’ interpretation of life engagement; the remaining PROs generally focused on a single aspect of life engagement, for example, the ability to feel pleasure. Overall, the results of this review indicate that there are no validated PROs that can capture all aspects of life engagement within the conceptual framework defined by expert psychiatrists [9], and thus it is not possible to fully evaluate the life engagement framework in research and/or clinical practice using existing tools.

Considering the two ‘life engagement’ PROs, the Engaged Living Scale (ELS) and Life Engagement Test (LET) each include items to measure motivation and reward, life fulfillment, and value-based living [22, 23]. The LET also includes an item to measure valued activities. However, neither PRO captures the cognitive or vitality aspects of life engagement, which are important aspects of treatment effectiveness [12]. Furthermore, both PROs are rather brief—the LET comprises just six items, and the ELS comprises six ‘life fulfillment’ items that measure a person’s current status (e.g., “I feel that I am living a full life”), with the remaining ten ‘valued living’ items being more conceptual and potentially less useful to track changes over time (e.g., “I know how I want to live my life”). Finally, both PROs appeared just once in eligible SLR articles, and neither PRO has been used in a sample with mental health disorders (though both negatively correlate with mental health symptoms), suggesting that neither PRO has become established in mental health clinical research or practice.

Considering specific aspects of life engagement, a number of widely used PROs were identified to measure pleasure–anhedonia, in line with a previous review in MDD [92]. Anticipatory pleasure (related to motivation to engage in rewarding and goal-directed behavior) and consummatory pleasure (momentary pleasure that is experienced while engaged in an enjoyable activity) may be suppressed in mental health disorders [93, 94], and are likely to be key components of life engagement. Other aspects of engagement for which commonly used PROs were identified included motivation/reward/energy–apathy, attention/alertness, mental/psychological well-being, life satisfaction/meaning, and rumination. However, none of these PROs encompass the full concept of life engagement.

Another observation from this review is that the majority of PROs appeared in the search results ≤ 3 times, indicating that most of these rating scales have not become established in research.

The implications of this review for research and clinical practice are that there is a need for a new, validated PRO to more comprehensively measure life engagement in mental health—a meaningful clinical outcome beyond improvement of core symptoms. Thus, future research should attempt to develop such a PRO, potentially by adapting or combining PROs identified in this review to better align with the framework. Recently, an expert panel attempted to repurpose an existing PRO of depressive symptoms to potentially capture multiple aspects of life engagement [95]. The panel selected relevant items from the Inventory of Depressive Symptomatology Self-Report (IDS-SR) to create the ‘IDS-SR_10_ Life Engagement’ exploratory subscale. Though in need of further validation and input from patients, the IDS-SR_10_ Life Engagement subscale has been used to show a benefit for active treatment versus placebo in a post hoc analysis of MDD clinical studies, and may represent a useful new tool in clinical research [95].

It should be noted that improvement in functional outcomes that reflect life engagement may lag behind symptomatic improvement, since the presence of unresolved mental health symptoms can limit improvements in other domains [96, 97]. Thus, a life engagement PRO may not show a treatment effect as quickly as a PRO that measures core mental health symptoms.

Limitations of this SLR include that a single database was searched; that a single reviewer selected articles, increasing the risk of rejecting relevant reports; and that PROs tend to be secondary or exploratory study outcomes in clinical trials, and thus may not be mentioned in the publication abstract. Finally, the conceptual framework of life engagement requires additional validation and further clarification on how it differs from existing concepts.

## Conclusions

Numerous PROs are available that may capture aspects of life engagement. However, a need remains for a new PRO that can be used in clinical trials in mental health disorders to provide a more comprehensive description of improvements in life engagement that may result from successful treatment. If such a tool can be developed, it will assist in the development of new treatments that may provide meaningful and valued benefits to patients with mental health disorders.

## Data Availability

All data generated or analyzed during this study are included in this published article.

## Abbreviations

ACT: Acceptance and commitment therapy
AES-S: Apathy Evaluation Scale Self-report
CIT: Comprehensive Inventory of Thriving
DARS: Dimensional Anhedonia Rating Scale
ELS: Engaged Living Scale
EMA: European Medicines Agency
FDA: Food and Drug Administration
IDS-SR: Inventory of Depressive Symptomatology Self-Report
LET: Life Engagement Test
MAP-SR: Motivation and Pleasure Scale—Self-Report
MDD: Major depressive disorder
MEI: Motivation and Energy Inventory
PRO: Patient-reported outcome
SACL: Stress Arousal Checklist
SLR: Systematic literature review
SMVM: Shirom–Melamed Vigor Measure
WHO-5: World Health Organization (Five) Well-Being Questionnaire
